# Evaluating the feasibility of “Friends in Nature,” a complex nature-based social intervention to address loneliness and quality of life in six cities worldwide

**DOI:** 10.1186/s40814-024-01575-4

**Published:** 2024-11-30

**Authors:** Cristina Vert, Jill S. Litt, Mireia Gascon, Marta Roqué, Montse Masó-Aguado, Nerkez Opacin, Gabriela Garcia, Anu Jansson, Lucie Cattaneo, Alžběta Bártová, Laia Briones-Buixassa, Aina Carbó, Laura J. Rautiainen, Laura Hidalgo, Ashby Sachs, Sara Domènech, Sergi Blancafort-Alias, Iva Holmerová, Kaisu H. Pitkälä, Laura Coll-Planas

**Affiliations:** 1 Barcelona Institute for Global Health (ISGlobal), 08003 Barcelona, Spain; 2 Universitat Pompeu Fabra (UPF), Barcelona, Spain; 3 CIBER Epidemiología y Salud Pública (CIBERESP), Madrid, Spain; 4 University of Helsinki, Department of General Practice, Unit of Primary Health Care, Helsinki University Hospital, Helsinki, Finland; 5 Department of General Practice, University of Helsinki and The Finnish Association for the Welfare of Older Adults, Helsinki, Finland; 6 Centre of Expertise in Longevity and Long-Term Care, Faculty of Humanities, Charles University, Prague, Czech Republic; 7 Research Group On Methodology, Methods, Models and Outcomes of Health and Social Sciences (M3O), Faculty of Health Sciences and Welfare, Centre for Health and Social Care Research (CESS), University of Vic-Central University of Catalonia (UVic-UCC), Vic, Spain; 8 Fundació Salut i Envelliment UAB, Casa Convalescència UAB C/Sant Antoni M. Claret, 171, 4a Planta, Barcelona, Spain; 9 Faculty of Architecture and Urbanism, University of Cuenca, Av 12 de Abril y Agustín Cueva, 010101 Cuenca, Ecuador; 10 School of Global, Urban and Social Studies, RMIT University, 124 La Trobe Street, Melbourne, VIC 3000 Australia; 11 Assistance Publique-Hôpitaux de Marseille (AP-HM), Marseille, France; 12 Biomedical Research Institute Sant Pau (IIB Sant Pau), Barcelona, Spain; 13 Institute for Research and Innovation, Life Sciences and Health in Central Catalonia (IRIS-CC), Vic, Spain; 14 Research Group On Innovation in Mental Health and Social Wellbeing (ISAMBES), Faculty of Health Sciences and Welfare, Centre for Health and Social Care Research (CESS), University of Vic-Central University of Catalonia (UVic-UCC), Vic, Spain

**Keywords:** Green spaces, Study design, Mental health, Feasibility, Group-based activities, Loneliness, Social prescribing, Nature-based interventions

## Abstract

**Background:**

Loneliness, a major public health concern, could be alleviated through social interventions with nature contact as a primary component. “Friends in Nature” is a complex nature-based social intervention designed to be implemented as part of “Reimagining Environments for Connection and Engagement: Testing Actions for Social Prescribing in Natural Spaces" (RECETAS). This project aims to alleviate loneliness and promote health-related quality of life in six different geographic areas worldwide. Feasibility studies are crucial to assess the viability of complex interventions and study procedures before conducting definitive studies. This paper aims to describe the design, implementation, and evaluation of the six-related feasibility studies on the “Friends in Nature” intervention. These studies specifically evaluate feasibility of recruitment and study procedures, intervention implementation, and data collection and distribution.

**Methods:**

We defined a comprehensive set of indicators to assess the feasibility of “Friends in Nature.” For the first domain, recruitment procedures were assessed to determine their adequacy, while attrition rates were examined to assess participant retention. For the second domain, the implementation of interventions was evaluated, along with the study design’s ability to adapt to unexpected situations and participant adherence to the intervention. Finally, for the third domain, completion rates and the acceptability of the study activities were also analyzed. The feasibility of using specific scales to assess loneliness and well-being was also explored.

**Results:**

The feasibility indicators defined for this study were useful to assess the feasibility of “Friends in Nature.” Recruitment procedures were generally found to be adequate, and the number of dropouts was low. Interventions were implemented with minor adjustments, and facilitators played a vital role in the well-functioning of the interventions. Although some unexpected situations occurred during the study, adaptations were made, and participants were generally satisfied with the activities proposed. Scales used to assess loneliness and quality of life showed potential for measuring the effects of nature-based social prescribing in the full trial.

**Conclusion:**

This paper offers valuable insights into the design and execution of feasibility studies for complex interventions like “Friends in Nature.” Findings from these assessments explore the feasibility of “Friends in Nature” and will inform the main RECETAS studies, which are designed to strengthen the evidence base to support the use of nature-based social prescribing to reduce loneliness and promote quality of life.

**Trial registration:**

Barcelona trial: NCT05488496, Prague trial: NCT05522140, and Helsinki trial: NCT05507684.

## Key messages regarding feasibility

- What uncertainties existed regarding the feasibility?

Uncertainties around feasibility encompass several key areas. First, it may be challenging to adapt the Circle of Friends intervention model to alleviate loneliness among older people in Finland to the “Friends in Nature” intervention, oriented around nature-based experiences and targeting other vulnerable population groups and diverse contexts. Second, it is unclear whether lonely individuals would be willing to enroll in and adhere to the study. Third, recruitment feasibility for at-risk participants across different demographics (lonely people of different age groups, gender, economic status, ethnicity, and cultural background) presents another concern. Fourth, there is also uncertainty around how well our intervention, “Friends in Nature,” could integrate into various care environments to support social prescribing. Fifth, the suitability of the selected primary and secondary outcome measures is another area of ambiguity. It is essential to confirm that these measures effectively capture relevant outcomes for diverse study populations. Finally, questions remain about whether the research methods and study assessment protocols are robust enough to support the evaluation of the “Friends in Nature” intervention.

- What are the key feasibility findings?

The Circle of Friends intervention model was successfully adapted to the target populations and circumstances across the study sites to develop the “Friends in Nature” intervention. Recruitment goals for different populations across study sites were met using tailored methods adapted to local conditions. To improve participant recruitment and retention, additional resources may be required to support tailored and on-the-ground recruitment efforts and monitor participant satisfaction over time. The engagement of lonely people with intervention components was high, and completion of study assessments was high (≥ 87%). The “Friends in Nature” intervention was conducted as planned, with some adjustments of time allocated for activities, mobility limitations among certain subpopulations, and adjustments to activity selection due to weather and cost of outdoor excursions. In one study area, intercultural briefings and one-to-one conversations were added to the first group sessions to avoid misunderstandings due to cultural differences. The research methods and study assessment protocols were sufficient to support the evaluation of the “Friends in Nature” intervention, although the health-related quality-of-life scale was not suitable in one community. The length of the questionnaire and size of font were flagged by some older adult participants. Although the feasibility study was limited by small sample sizes assessed at baseline (T1) and follow-up (T2) timepoints, they suggest that the quality of life and loneliness scales had adequate responsiveness to change across the sites and would be appropriate to measure the effect of nature-based social prescribing (NBSP) on quality of life and loneliness in the main evaluative studies.

- What are the implications of the feasibility findings for the design of the main study?

The feasibility findings suggest that more extensive randomized controlled and pre-post studies are viable to study the impact of the “Friends in Nature” intervention in reducing loneliness and improving health-related quality of life. The intervention is likely/expected to be well-received by lonely people included in the main studies. Enrollment and study assessment completion rates were sufficient to support full-powered randomized trials and pre-post studies. Selected measures are sensitive and likely to capture changes of the intervention relative to a control arm or baseline comparison in the definitive studies. The results underscore the importance of co-creation and stakeholder engagement to support recruitment and intervention implementation at the neighborhood level.

## Background

Loneliness stems from a disparity between the quantity and quality of social relationships one has and those that are desired. The perception of feeling lonely can occur despite being surrounded by people [1]. Loneliness has emerged as a significant public health concern [2–4] which not only affects mental well-being but also has tangible effects on physical health, such as increased morbidity and mortality rates. Chronic diseases like cardiovascular disease, type 2 diabetes, and cerebrovascular disease have been associated with loneliness, along with conditions like anxiety, depression, cognitive decline [5, 6], and mental well-being [7].

Nature-based interventions are believed to foster social connections and reduce the risk of loneliness [8]. Nature-based social prescribing (NBSP) is a social prescribing program [9, 10] that incorporates nature-based activities and social support to improve health and well-being. NBSP is an innovative socio-environmental approach that offers a non-medicalized intervention that can be integrated into healthcare systems to address loneliness and other conditions affecting mental health and well-being more effectively. This intervention includes access to nature as a primary component and can be designed to emphasize group-based activities.

Social prescribing is being applied following different models, including one that has been recently described as holistic and group based [11]. This holistic, group-based model serves as the foundation for the newly designed “Friends in Nature” NBSP intervention. “Friends in Nature” is a social intervention adapted from the Circle of Friends® methodology [12] and emphasizes nature-based activities, with an overarching aim of alleviating loneliness and enhancing health-related quality of life across different age groups and diverse geographic areas.

**Fig. 1:**
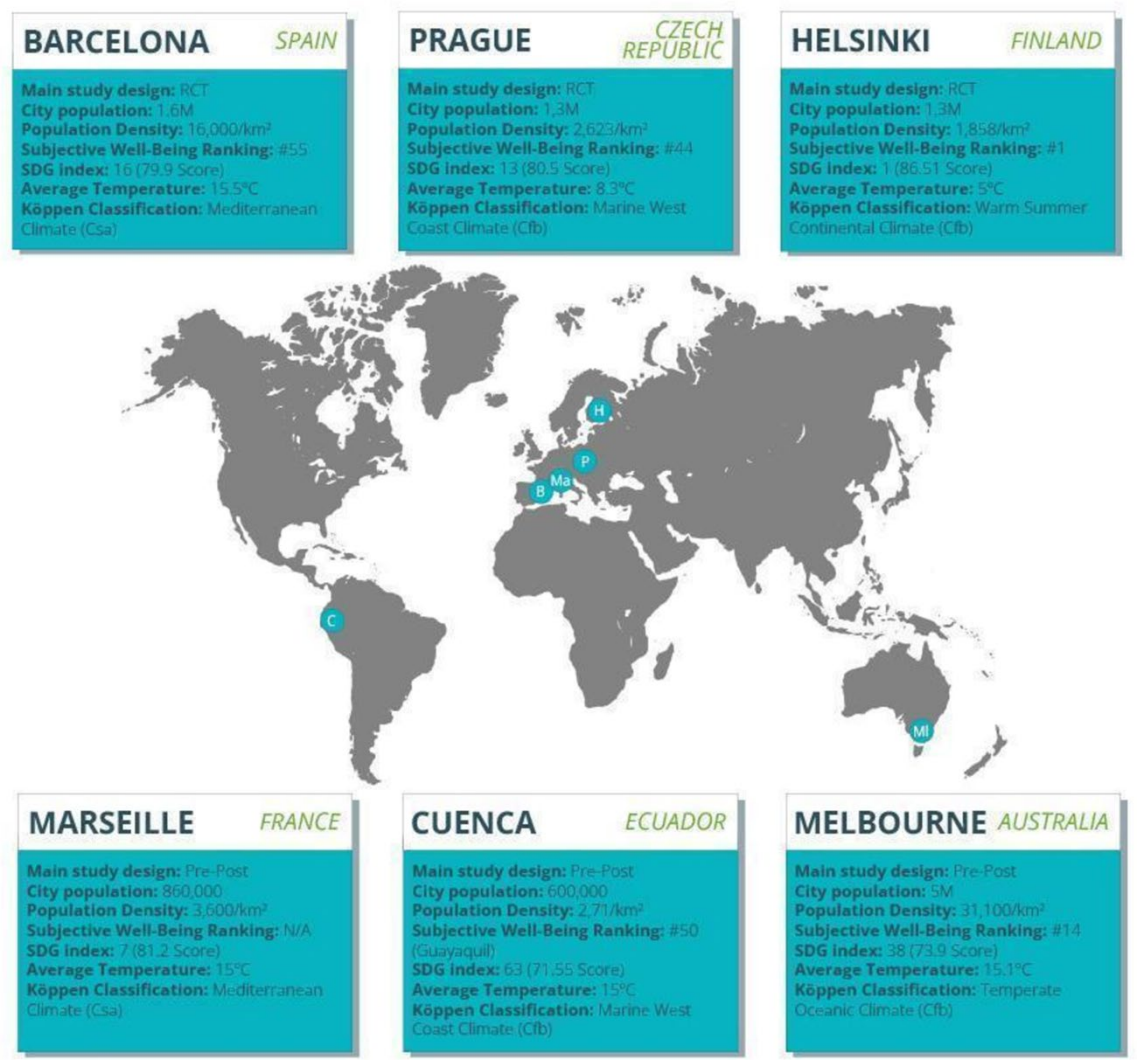
Selected cities for the study. RCT, randomized controlled trial. M, million. SDG, Sustainable Development Goal

The “Friends in Nature” intervention is being developed and implemented as part of “Reimagining Environments for Connection and Engagement: Testing Actions for Social Prescribing in Natural Spaces” (RECETAS) [13], a 5-year research project funded by the European Union’s Horizon 2020 research and innovation program. The project aims to explore the effectiveness of interventions to support NBSP in the alleviation of loneliness and the enhancement of health-related quality of life of vulnerable groups in six cities across the globe, including Melbourne (Australia), Barcelona (Spain), Helsinki (Finland), Marseille (France), Prague (Czech Republic), and Cuenca (Ecuador) (Fig. 1). This will be achieved in the definitive studies through randomized controlled trials (RCT) in Barcelona, Helsinki, and Prague and pre-post intervention studies in Melbourne, Marseille, and Cuenca, to be implemented for an expected duration of 10 weeks in each site [14]. Thus, according to the definition of the Medical Research Council [15], “Friends in Nature” is considered a complex intervention because (i) it targets different vulnerable population groups and is tailored to its different characteristics (see Table 1), (ii) it is to be implemented in diverse cultural contexts in different cities from various continents — as described above — and (iii) its evaluation involves multiple health outcomes and both quantitative and qualitative methods.

**Table 1:** Community organization and target group characteristics in each intervention site

City (intervention site)	Community organizations	Specific characteristics of the target group of participants
Helsinki	Assisted living facilities	Older adults (55 +) in assisted living facilities
Prague	Organizations of seniors, local authorities, social care centers, primary care providers	Older adults (60 +) with risk of social isolation and loneliness living in the community
Barcelona	Primary care centers, social services centers, and civic organizations	Adults (18 +) living in socio-economically deprived urban areas
Cuenca	Organizations serving older adults and nursing homes^a^	Older adults (65 +) living alone or in nursing homes, within the urban area of Cuenca
Marseille	Social services centers, housing, and social rehabilitation centers	Adults (18 +) living in socio-economically deprived urban areas
Melbourne	Organization which provides support to LGBTIQA + (lesbian, gay, bisexual, trans, intersex, queer, asexual, other diverse sexual orientations, and gender identities) refugee and asylum seeker communities	Adults (18 +) who belong to the LGBTIQA + refugees and asylum seekers or LGBTIQA + people from culturally and linguistically diverse communities

^a^In Cuenca, participants were recruited in two different community organizations: a nonresidential [Centro Municipal de Cuidados del Adulto Mayor (CCAM)] and a residential center for older adults [Hogar Cristo Rey (HCR)]

Before conducting the abovementioned main studies to understand the effects of a complex intervention — “Friends in Nature” — on reducing loneliness and promoting health-related quality of life, it is necessary to conduct feasibility studies. Assessing feasibility is necessary to increase certainty for the proposed intervention among diverse vulnerable populations in different geographic contexts related to the following: (1) participating in an intervention about loneliness; (2) framing the study to reach different populations at risk (e.g., different age groups, gender, economic status, ethnicity, cultural background); (3) positioning the study to fit within different care environments to support social prescribing; (4) testing appropriateness of primary and secondary outcomes for these populations; and (5) piloting research to evaluate our research aims. Therefore, this paper aims to describe the design, implementation, and evaluation of the six-related feasibility studies on the “Friends in Nature” intervention. These studies followed a well-planned feasibility study protocol that was conceptualized as part of the initial planning and design of the intervention study [13]. Specific objectives of the six-related feasibility studies were to assess the following: (i) the feasibility of recruitment and study procedures, (ii) the implementation of the intervention, and (iii) the acceptability and quality of data collection and data distribution.

## Methods

### Study design

We conducted six-related feasibility studies to determine whether and how “Friends in Nature,” the main intervention proposed by the RECETAS project, could be conducted. In five intervention sites (all except Barcelona), feasibility studies were designed as noncontrolled exploratory pre-post intervention studies. For Barcelona, the local team decided to test randomization with a control arm. The six-related feasibility studies employed a mixed-methods design, with quantitative and qualitative assessments, and lasted between 4 and 9 weeks, depending on the site. This paper is focused on the quantitative evaluation.

### Study population

#### Sample size

The sample size used in the feasibility study was smaller than the sample size for the full study. A sample size calculation is not required for feasibility studies [16, 17]. However, for the purposes of the feasibility study, we aimed to recruit between 6 and 12 participants in the treatment arm per site to create the group dynamic needed to test the implementation of the intervention elements. In Barcelona, we aimed to randomize 1:1 in order to test the randomization procedures.

#### Recruitment methods

Recruitment procedures were adapted to local contexts for each intervention site. Although there were common principles followed by each site, each local research team worked closely with community organizations from where participants were approached, screened, and finally selected. Community organizations included assisted living facilities, centers for older adults, social centers, residences, or apartment complexes — among others — hosting a diversity of population groups (Table 1).

In Helsinki, Cuenca, Barcelona, and Marseille, the research team visited the community organizations to introduce the details of the feasibility study to potential participants, who contacted the research teams when interested in participating. Additionally, in Marseille, the research team published information online and distributed flyers and posters in the community organizations, suggesting to the professionals encountered to redistribute them to other professionals or individuals potentially interested in the study, thus creating a snowball effect.

In-person visits were not part of the recruitment strategy in Prague. The research team in Prague sent letters and leaflets to potential participants previously identified by the partners of the community organizations and municipal authorities. They also advertised the feasibility study in the local newspaper and on the radio. The participants interested in the study contacted the research team.

In Barcelona, community organizations that were already implementing a social prescribing program collaborated with the study. The range of strategies used to recruit participants included the use of social media (Facebook, Twitter, and Instagram), print media, and online and face-to-face information sessions. Additionally, the research team asked stakeholders and partners of the community organizations to post information about the program on their websites, mailing lists, blogs, and social media sites. The municipality’s social services and primary care organizations were also involved in the feasibility study and referred individuals to the program. To strengthen the recruitment in Barcelona, researchers met with all involved stakeholders and reinforced visits in the neighborhood (senior housing, charity support organizations, grocery stores, and pharmacies).

Finally, in Melbourne, recruitment occurred through multiple channels. A flyer calling for participants was circulated via email and distributed through a WhatsApp group administered by the community organization engaged with the recruitment of participants in this intervention site. Also, the pilot study was promoted through in-person events and direct personal contact.

In all the intervention sites, individual meetings between the research team and the potential participants were organized to check, by completing a checklist, whether the participants met the inclusion criteria. Participants selected for the feasibility study were provided with an information document with understandable vocabulary and signed the informed consent. Also, in assisted living facilities in Helsinki, the participants’ cognitive state was measured by using the Clinical Dementia Rating (CDR) [18], and those with moderate dementia needed the informed consent also from their closest proxy.

#### Participant eligibility

General inclusion criteria for the eligibility of participants of the six-related feasibility studies were as follows:

- Participants should be able to understand informed consent in the corresponding local language: English, French, Catalan or Spanish, Czech, or Finnish.
- Adults or older adults (see Table 1 for specific cutoffs).
- Screened positive for loneliness: This was assessed with the screening question “Do you suffer from loneliness?”. From a 5-point scale [from 1 (never) to 5 (always)], participants met the inclusion criteria when they answered “3. Feels lonely sometimes,” “4. Often,” or “5. Always” [19].
- Willing to undergo study measurements and engage in nature-based activities during the study

Exclusion criteria included the following:

- Unable to go outdoors due to poor mobility or severe diseases
- Poor hearing or sight: This criterion did not apply in Cuenca.
- Mild, moderate, and severe cognitive decline in all cities except Helsinki where only people with moderate and severe cognitive decline were excluded when the mini-mental state examination (MMSE) < 15 [20].
- Severe disease with poor prognosis < 6 months

However, apart from the inclusion criteria applicable to all the participants, there were some specificities that characterized the study population in each intervention site (Table 1).

### Interventions

#### Facilitator’s training

The implementation of “Friends in Nature” required specific training to prepare health or social care professionals as facilitators. Facilitators were an essential asset to guarantee the correct functioning of the interventions. Using a modified training model of the Circle of Friends® methodology [21] developed by the Finnish Association for the Welfare of Older Adults and the University of Helsinki, RECETAS project partners organized online or in-person workshops during the feasibility study period to train facilitators. This training model was specifically adapted to nature-focused adding academic content on how nature enhances wellbeing and conducting some of the experiential dynamics in a natural setting. Moreover, each site adapted the knowledge and expertise gained through the training to its sociocultural context and target population to better reflect the needs of the local population. Specifically, during the co-creation process, stakeholders also contributed to identifying the specific profiles, needs, and characteristics of the target population on the corresponding site. This information allowed sites to adapt the training content with cases and examples to the target population reached by each RECETAS site, with its socioeconomic and cultural characteristics. Finally, as the main element of the intervention, trained facilitators supported the group process and group dynamics fostering empowerment and autonomy.

#### Design of the intervention

The “Friends in Nature” intervention has two main components that are expected to complement and make synergies with each other: (1) peer support group and empowerment process including specific group dynamics and elements that were adapted according to the Circle of Friends® methodology [12] (individual interview, empowerment letter, learning diaries, and training) and (2) nature-based activities chosen by participants, aligned with their preferences and based on a NBSP menu [14]. Trained facilitators are key personnel in the intervention, as described in the previous section of this paper.

Specifically, the “Friends in Nature” approach aims to create a supportive network around vulnerable individuals suffering from loneliness to promote their inclusion, social integration, and overall well-being in their community. By fostering positive relationships and social connections, this approach aims to enhance the individual’s self-efficacy, social skills, and overall quality of life. Participants in each intervention site have available NBSP menu built through participatory methodology by the RECETAS team project, in which a diversity of NBSP activities are initially offered [22], and participants can also propose activities. The menu includes activities promoted by the municipality or grassroots organizations, which can accommodate the group of participants, open and freely accessible nature areas, or new activities specifically organized for the RECETAS group. It varies among sites, but it usually includes outdoor physical activities (e.g., nature walks, tai chi, yoga, stretching), nature-based observational activities (e.g., bird watching, wildlife observation, pet interactions, forest bathing), tactile-related activities (e.g., gardening, farmers market), and arts and nature activities (e.g., urban sketching, nature in museums and arts, virtual nature experiences). The latest — always with the component of nature — are also usually included on the menu in case it is not possible to go outdoors.

In all sites, the first group-based session is planned indoors. Participants get to know each other, agree on the basis for participation in the group (such as respect and confidentiality), and discuss the different activities offered in the NBSP menu [22] to finally choose one or more activities to be conducted in the following sessions. Activities can also be directly suggested by the participants. However, this selection is flexible and adaptable over time, depending on the participants’ preferences and local needs. The remaining sessions are encouraged to be conducted in natural spaces outdoors as much as possible, which depends on the weather and mobility levels. Participants are engaged in the chosen activity. Sessions take place once a week for approximately 2 h (including trips).

##### Control arm

In Barcelona, which was the only study site with a control arm, participants in the control arm were given the menu with the list of nature-based activities and other resources in their environment as a resource sheet and underwent an individual interview with the facilitators to know their needs and preferences and set recommendations on which activities would fit them best. It is considered a sign-posting model of social prescribing [11]. In this case, participants were not organized into groups and could choose individually whether to undertake the activities.

### Feasibility indicators

In order to assess feasibility, we identified three domains related to recruitment, implementation, and data collection. Domain 1 included recruitment and attrition; Domain 2 included implementation, adherence, and adaptation; and Domain 3 included completion rate, acceptability, and variable distribution and variability. These are described in Table 2. These indicators selected to assess the feasibility of “Friends in Nature” are based on those suggested in the extension of the CONSORT (Consolidated Standards of Reporting Trials) 2010 statement for randomized pilot and feasibility trials [23, 24].

**Table 2:** List and description of the feasibility indicators evaluated in the feasibility study by each intervention site

Feasibility indicator	Description
Domain 1*: *Assess the feasibility of recruitment and evaluate study procedures	
** Recruitment**	• Number and percentage of eligible participants contacted, screened, and consented • Time needed to recruit the participants required for the feasibility study in each intervention site
** Attrition**	• Number and percentage of participants who dropped out of the feasibility study • Their reasons for doing so
Domain 2*:* Assess the implementation of the intervention	
** Implementation**	• Assessment of the likelihood and the manner the intervention could be fully implemented as it was designed and proposed (e.g., assess whether the time allocated for each activity and measurement is adequate) • Assessment of the reactions and satisfaction of the participants with the interventions and the activities proposed in the NBSP menu • Recommendations for improving intervention for the main study
** Adherence** (of the participants to the intervention)	• Number of sessions attended, including trends in attendance over time • The reasons for attendance or nonattendance • Participants’ willingness to continue with any of the activities conducted during the study
** Adaptation**	• Assessment of unexpected situations that arise during the intervention and the modifications conducted to adapt to them
Domain 3: Evaluate data collection and distribution	
** Completion rate** (quality of the data)	• Number of fully completed questionnaires among the total number of initiated questionnaires, as well as the number of missing values
** Acceptability**	• Participants’ reactions towards interviews/questionnaires regarding factors such as readability, comprehension, balance, fairness, and interest • Assessment of the time required to respond to the questionnaires/interviews • Reactions to measurement tools
** Variables distribution** and **variability**	• Distribution and variability of the variables collected to assess loneliness, quality of life, and cost-effectiveness outcomes for a specific sample

#### Outcome assessments

As part of the assessment of the feasibility indicators from Domain 3, the feasibility studies tested the data collection procedures to be used in the corresponding main studies of the RECETAS project, considering all or a subset of the outcomes planned, to assess completion rate and acceptability.

An initial pre-intervention assessment (T1) was conducted before the start of the intervention with a one-to-one session where participants were interviewed individually by the outcome assessors. A questionnaire assessed two primary health outcomes of the six-related feasibility studies: loneliness and health-related quality of life. Loneliness was measured by an interviewer using the 11-item De Jong Gierveld (DJG) Loneliness Scale [25], and health-related quality of life was measured using the 15-dimensional measure of health-related quality of life (HRQOL-15 D scale) [26]. Questions about the participants’ interests in nature and suggestions regarding group-based activities were also included in the T1 assessment. A post-intervention assessment (T2) to assess participants’ loneliness and health-related quality of life was conducted after the intervention. It also included questions to assess the reactions and satisfaction of the participants with the intervention and the activities conducted and recommendations for improving. Moreover, for the post-intervention, facilitators also answered questions to qualitatively assess the implementation of the intervention, i.e., whether the intervention could be fully implemented as it was designed. Finally, after each nature-based activity, a nature dose questionnaire was given to the participants in some intervention sites. A specific questionnaire was developed to capture different elements that allow the evaluation of the dose of nature received by the participants after each session.

Additionally, during the feasibility study, trained facilitators reported all the potential setbacks experienced and how these were addressed to undertake the activities embedded in the intervention, and outcome assessors reported the time required to respond to the questionnaires among other feasibility indicators detailed in Table 2.

### Randomization

For the feasibility RCT in Barcelona, after the baseline assessment, and once the consent of the interested participants was obtained, researchers subdivided the sample of participants into two groups: the intervention arm and the control arm. Groups were randomly assigned using a predefined computer-based block randomization scheme, with randomly varying block sizes. The project statistician generated random assignments for each participant. Allocation of participants was concealed, with the statistician disclosing the allocation to the intervention arm only after each participant was included in the study, assigned an identification code, and completed the study baseline assessment. All assessments were unblinded for the feasibility studies.

### Data management

A data management plan was prepared for all sites, and its implementation was assessed in terms of data collection methodology and standards, data storage security, and data confidentiality. The methods and instruments used for data management varied across intervention sites. Descriptive and outcome data were collected with paper questionnaires in all sites, although in Barcelona the REDCap (Electronic Data Capture system) was used for post-intervention assessments. In Helsinki, qualitative interviews and focus-group discussions were recorded and written verbatim. In groups where participants suffered from dementia, the field notes of facilitators were essential for assessing feasibility. Data was stored electronically in REDCap in Helsinki and Barcelona and in a local secure server in Prague, Melbourne, and Cuenca.

### Data analysis

The two primary outcomes to be analyzed included DJG loneliness scale and the HRQOL-15 D scale. Total scores for these scales were computed following original papers [25, 26]. The health-related quality single HRQOL-15D score reported on a 0–1 scale and was presented as percentages for ease of understanding. Descriptive analyses were conducted, calculating frequencies of categorical outcomes, and medians, as a measure of central tendency for the primary outcomes and feasibility indicators assessed in this study. For scales that had a categorization, an overall score and categories were created and tabulated.

## Results

The following sections detail the results of the feasibility studies for five of the six cities (Barcelona, Cuenca, Helsinki, Melbourne, and Prague), and results are reported in Table 3. The feasibility study in Marseille was stopped because many of the participants could not attend the sessions within the expected timeframe. Therefore, the results from this intervention site were not included in this evaluation.

**Table 3:** Summary of the results of the feasibility studies in each intervention site and for each feasibility indicator

	**Barcelona**	**Cuenca**	**Helsinki**	**Melbourne**	**Prague**
**Domain 1: Recruitment and attrition**					
**Recruitment**					
People contacted, screened, and consented (n)	26, 19, 15	CCAM: 23, 22, 9 HCR: 21, 13, 7	7, 7, 6	N/A, N/A, 6	28, 14, 14
Participants eligible for the feasibility study (%)	79%	CCAM: 39% HCR: 33%	88%	100%	50%
Eligible men (%)	20%	CCAM: 33.33% HCR: 85.71%	33%	N/A	7.14%
Recruitment period (number of days)	59 days	CCAM: 15 days HCR: 15 days	11 days	305 days	24 days
**Attrition**					
Dropouts (n)	6 (2 in intervention and 4 in control arm)	0	0	0	1
**Domain 2: Implementation of the intervention**					
**Implementation**					
Time allocated for sessions	More than 2 h needed sometimes	Around 2 h	Occasional abbreviation of sessions due to participant fatigue	Usually more than 2 h were needed	Perception of excessive time allocated to activities
Responses to intervention	Happy to participate	Visual impairment and/or hearing difficulties were a barrier for some participants to fully enjoy the sessions	No complaints	Very positive response to the intervention	Participants were satisfied with the intervention
Learning diary/field notes	Yes	Yes	Yes	Yes	Yes
**Adherence**					
Adherence rate (%)	38–75%	54–100%	83–100%	50–100%	64–71%
Participants’ willingness to continue (n)	5	-	6	-	13
**Adaptation**					
Adaptation to unexpected situations	Adjustment of group sessions due to low attendance	Accommodation to weather conditions and emotional and behavioral conflicts	One participant waiting several hours with the facilitator	Introduction of intercultural briefing and group conversations to avoid potential misunderstandings	No unexpected situations
**Domain 3: Data collection**					
**Completion rate**					
Completion rate and time required to respond T1 (%, minutes)	100%, 90 min	CCAM: 100%, 45 min HCR: 100%	0% (no permission from Helsinki city at the time)	100%, 45 min	100%, 45 min
Completion rate and time required to respond T2 (%, minutes)	50%, 60 min	CCAM: 100%, N/A HCR: 85.7%	100%, 90–120 min	50%, N/A	100%, N/A
Missing data (name of the questionnaire: specific question that led to missing data)	None	HRQOL-15 D: question 15 on sexual activity	HRQOL-15 D: question 15 on sexual activity	DJG: question 8 on loneliness	HRQOL-15 D: question 15 on sexual activity
**Acceptability**					
Complaints about the questionnaire	No complaints	Length of the questionnaires	No complaints	Complaints about HRQOL-15 D questions (vocabulary, inaccuracy)	Length of the questionnaires
**Variables distribution and variability**					
Median quality of life (HRQOL-15 D) at T1 (%)	88%	74%	68% (T2)	91%	93%
Median absolute change of quality of life after intervention (T2) (% of change)	6% improvement	n/a	n/a	3% decrease	2% decrease
Median loneliness (DJG) at T1	7	9	7 (T2)	4	6.5
Median absolute change of loneliness (DJG) after the intervention (T2)	0	3	N/A	0	0.5

*N/A* not available. *CCAM* Centro municipal de Cuidados del Adulto Mayor. *HCR* Hogar Cristo Rey (these are the names of the two community organizations where the intervention was conducted in Cuenca, Ecuador. The feasibility study in Cuenca was conducted in two different community organizations). *T1* baseline assessment. *T2* follow-up/post-intervention assessment. *HRQOL-15 D* Quality-of-Life Questionnaire. *DGJ* De Jong Gierveld Scale (to assess loneliness). Note: Barcelona was the only intervention site where randomization was conducted. However, only one participant completed the study in the control arm (out of five participants, because four participants left). Therefore, results for the control arm are not shown in Table 3

### Domain 1: Recruitment and attrition

**Fig. 2:**
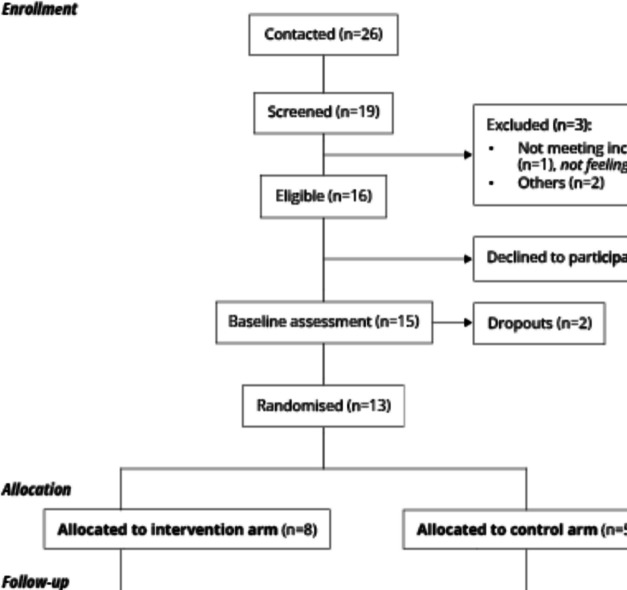
Barcelona Feasibility Trial Profile

The results of the feasibility studies for recruitment generally matched the target estimates defined in the feasibility study protocol. The number of people contacted, screened, and consented was very similar in Barcelona [26, 19, and 15] (Fig. 2) and Prague [28, 14, and 14] and lower in Helsinki [7, 7, and 6] and Melbourne [13, 8, 8]. In Cuenca, participants were recruited in two different community organizations: a nonresidential and a residential center for older adults. In both cases, 23 and 21 participants were contacted, 22 and 13 were screened, and 9 and 7 were consented. The lowest percentage of participants eligible for the feasibility study was found in Cuenca [33%], whereas this percentage ranged between 50 and 100% in the other intervention sites. This is explained because some participants in Cuenca were not eligible because they did not meet the inclusion criteria (cognitive impairment or not feeling alone) or were not interested in the study. The percentage of eligible men recruited per site showed a wide spectrum, from 7% in Prague to 86% in the nursing home facilities for older adults in Cuenca. Values were more similar among each other in the other intervention sites: 20% of eligible men in Barcelona and 33% in Helsinki and in the community dwelling in Cuenca. These data were not available in Melbourne.

The recruitment period ranged from 10 days in Helsinki, to around 1 month in Prague, Cuenca, and Melbourne, and 2 months in Barcelona. In some intervention sites, the screening question for loneliness was not directly asked to participants because partners approached and screened people that they already knew were suffering from loneliness. The information given to participants during the recruitment seemed to be adequate, and only a few people contacted the research team to ask questions about the study. Most of these questions were related to schedules and activities. Some challenges arose during the recruitment phase in Barcelona related with the identification of people suffering and admitting loneliness, the social vulnerability and commitment of potential participants, and the high volume of work assumed by some community organizations.

The number of dropouts during the study are described below (Fig. 2 and Table 3). Reasons for dropping out were the decision to not participate in the study, lack of interest, health-related problems, or finding a new job.

The length of the feasibility studies ranged from 4 to 9 weeks^1^ in the different intervention sites.

### Domain 2: Implementation of the intervention

The assessment of implementation revealed that the interventions could be conducted as planned in all sites, with some adjustments. First, time allocation for activities was a common challenge in different intervention sites. While the Helsinki team occasionally had to abbreviate the sessions due to participant fatigue and the Prague team perceived the allocated time for the activities to be excessive, it happened to be the opposite in the other intervention sites. For example, outdoor group-based sessions were intended to last 2 h, including trips. Nevertheless, in Melbourne, sessions lasted up to 4 h plus 1–1.5 h for trips. In Cuenca, sessions lasted 2–2.15 h, including trips no longer than 30 min. And many sessions in Barcelona lasted 2.5–3 h.

Moreover, in Barcelona, the low number of participants in some sessions made it difficult to organize some of the activities. However, this challenge is not expected to be a concern in the main study, and efforts will be put to reach larger group sizes. Second, participants had generally positive reactions to the interventions, and they were enthusiastic about nature-based activities. Only a few participants in Cuenca with visual impairment and/or hearing difficulties did not always follow the thread of the conversations, and it was more difficult for them to fully enjoy the sessions.

During the feasibility study, facilitators in Cuenca, Helsinki, Melbourne, Prague, and Barcelona completed a learning diary or wrote field notes. Also, the trained facilitators received mentoring and feedback from expert trainers from Finnish Association for the Welfare of Older Adults, which was helpful in improving the management of sessions and group results. Overall, clear, and continuous communication with trained facilitators was seen as important for successful implementation.

The adherence rate (i.e., proportion of participants attending the sessions) in Barcelona ranged between 38 and 75% in the intervention arm, and it was 20% in the control arm (data not shown). It ranged between 50 and 100% in Melbourne, between 64 and 71% in Prague, and between 54 and 100% in Cuenca. Finally, in Helsinki it was 100% in 7 out of the 9 sessions. Reasons for not attending the sessions were diverse (e.g., medical issues, lack of interest, conflicting appointments, and care of a family member). Nevertheless, participants generally showed interest and willingness to continue with any of the activities conducted during the feasibility study.

The assessment of adaptation revealed that some unexpected situations were encountered during the intervention in all the sites, but modifications were made to overcome them. In Barcelona, facilitators adjusted group dynamics due to the low attendance in some sessions, and activities were adapted to the mobility capacity of the group. In Helsinki and Cuenca, group facilitators adapted activities to accommodate unexpected situations such as adverse weather conditions. In Finland, due to inclement weather and the high cost of outdoor excursions, some sessions had to be conducted indoors. Nonetheless, all sessions focused on the theme of nature, even if conducted indoors. The research team in Cuenca had to deal with a participant suffering an emotional crisis and another participant causing behavioral conflicts, both kept in the study after successfully dealing with the situation. In Melbourne, an intercultural briefing and one-to-one conversations were added to the first group-based session to avoid potential misunderstandings due to cultural differences. Finally, the research team in Finland could not conduct the T1 assessment due to a delay in getting the ethical approval.

### Domain 3: Data collection and variability

The completion rate for T1 was between 94 and 100% in all the intervention sites and between 50 and 100% for T2. Cuenca, Helsinki, and Prague experienced higher rates of missing data in questions from the HRQOL-15 D scale related to sexual activity, while Melbourne had more missing responses for questions about loneliness (from the DJG questionnaire). In Barcelona, missing data were more prominent in questions assessing secondary outcomes about neighborhood attachment, aesthetics, and participation in group activities.

Overall, the participant reactions towards interviews/questionnaires were positive. Nevertheless, the time required to complete the questionnaires differed among intervention sites. The T1 assessments in Barcelona were initially self-administered on paper, requiring 90 min per person for completion. However, due to the lengthy process, it was decided to switch to a more efficient approach using a guided one-on-one interview. The assessments were then administered directly through REDCap, a secure, web-based software, resulting in a reduced time of 45 min per person, which was comparable to the time taken in Prague.

In Melbourne, some scales (e.g., the HRQOL-15 D scale to assess health-related quality of life) were not suitable for the study population. Participants considered that the questions were not relevant, inappropriate for the age group, or too difficult to understand because of the vocabulary used which was considered to be inappropriate. In Cuenca, some participants complained about the length of the questionnaire and the font size, while in Prague the interviews/questionnaires were considered adequate and understandable although too long for some participants.

The sensitivity to changes before and after the intervention of the variables collected to assess the main study primary outcomes (i.e., the HRQOL-15 D scale [26] to assess health-related quality of life and the 11-item DJG scale [25, 27] for the assessment of loneliness) were assessed for each site**.** Participants in Barcelona, Prague, Cuenca, and Melbourne had moderate to high average quality of life measured with the HRQOL-15 D scale at T1 (median 88%, 93%, 74%, and 91%, respectively). Participants in Helsinki were only assessed at T2, with moderate quality of life (68%). On average, participants showed little change in quality of life after the intervention (Barcelona: a median absolute improvement of 6%; Prague and Melbourne: median absolute decreases of 2% and 3% respectively). However, at individual level, the scale was able to reflect the spread of individual responses to the intervention, with changes in quality of life ranging from important decreases (up to 22% absolute decrease) to important increases (up to 21% absolute increase).

The DJG scale has a range of values from 0 (meaning the individual does not feel lonely at all) to 11 (meaning the individual feels extremely lonely). The participants in Melbourne had the lowest levels of loneliness at T1 (median DJG scale value of 4), while participants in Barcelona and Prague had moderate loneliness (median DJG at T1 of 7 and 6.5, respectively) and participants in Cuenca had the severe levels of loneliness (median DJG at T1 of 9). On average, there were little to no changes in loneliness after the intervention (median absolute change of 0, 0, and 0.5 points in Melbourne, Barcelona, and Prague, respectively), except for Cuenca, where a relevant improvement in loneliness was observed (median change in DJG of 3 points). However, the scale was able to reflect large individual improvements in loneliness in response to the intervention in Barcelona, Prague, and Cuenca (up to 8-point change).

While these results are limited by the small sample sizes assessed at T1 and T2, they suggest that the HRQOL-15 D and DJG scales have adequate responsiveness to change across the sites and are appropriate to measure the effect of NBSP on quality of life and loneliness in the main studies of the RECETAS project.

## Discussion

This paper describes the feasibility of the “Friends in Nature” intervention, a complex nature-based social intervention aimed at alleviating loneliness and promoting health-related quality of life among vulnerable groups in six urban areas across Europe, South America, and Australia. These findings will inform the main RECETAS studies in these respective cities. Moreover, this paper provides valuable insights into the design of feasibility studies for other complex interventions, outlining the definition of appropriate indicators to evaluate the feasibility of study procedures, implementation of interventions, and the methods used for data collection and analysis.

### Main findings

The feasibility indicators established for this study were useful to explore the three key domains of feasibility of recruitment and study procedures, intervention implementation, and data collection and distribution. Recruitment procedures were generally adequate, except in Marseille, where the definition of the target population was deemed insufficient. This led to inadequate recruitment and prevented the feasibility study from progressing as planned at the intervention site. The team in Barcelona experienced more challenges, and some adjustments were needed. The key to the success was not the length of the recruitment, or the methods used to advertise the study, but rather the engagement with community organizations where the recruitment was conducted. Also, it was important to select an adequate community organization where the target population could be approached and recruited. This was shown in Cuenca, where some challenges were faced because the number of eligible participants was low.

Attrition results exceeded expectations, showing a low number of dropouts among intervention participants, with the highest percentage of dropouts being in the control group in Barcelona. Improvements to increase retention of controls included the reframing of the intervention options to include a group intervention and an individual intervention for controls, which included an individual interview, the nature-based activities menu, and other related information plus in-person health visits. Gender balance should be considered during recruitment. The results of the feasibility studies showed a non-balance between the men and women recruited per site, which can lead to biases in the results or challenges in implementing the intervention. As an example, in the feasibility study conducted in Prague, one participant dropped out because he was the only man in the group and reported not feeling comfortable in a group of only women.

The intervention “Friends in Nature” was generally implemented as designed in the feasibility study protocol, with just a few adjustments. A common challenge faced in different intervention sites was the time allocated for the activities. In those cases, sessions were adapted to the time needed to conduct the activities. While the core intervention elements worked well, and participants’ reactions to the interventions were generally positive, some participants found the activities to be too demanding or too time-consuming.

Some strategies were adopted to improve intervention implementation (see considerations for the main study below). For example, in Melbourne, the sessions started with a meal with the participants, which facilitated group reflection and conversations during mealtimes. Additionally, the team established a group social network via WhatsApp, which helped participants stay connected and informed of potential changes. The chat was also used to share thoughts and pictures from activities. Moreover, the participants who were not able to attend a particular session still felt as if they were part of the group experience.

In addition, trained facilitators were important assets for the well-functioning of the interventions. With their unwavering motivation, they not only accompanied and guided the participants throughout the intervention but also fostered an environment of comfort and positivity. The reactions of the participants towards the facilitators were very positive.

The results of the assessment of adherence of participants to the intervention were heterogeneous among the intervention sites, including the reasons for not attending the sessions. Nevertheless, participants generally showed interest in continuing with the activities conducted during the intervention.

Indicators of adaptation showed that even though different unexpected situations occurred during the study, these could be handled with minor modifications to the study procedure.

The completion rate of questionnaires for T1 was higher than for T2. Participants’ reactions towards the interviews and questionnaires were generally positive, although the time needed to answer them differed across intervention sites. Also, some participants found the assessment to be too long, and some questions considered to be inappropriate and sometimes unnecessary. The scales used to assess the primary health outcomes of the feasibility studies (DJG and HRQOL-15 D) were found to be too complicated for some participants to understand. This was the case for the participants in Cuenca and Prague, where the study population is over 65 years old. Despite these concerns, the overall assessment of the HRQOL-15 D and DJG scales suggested that they may have adequate responsiveness to change and will be appropriate tools to measure the effect of NBSP in the full trial.

Finally, although a webinar and standardized template were provided to each site to guide the pre-specification of estimated criteria for feasibility indicators, not all sites were able to complete this at the required time due to unknown factors for some indicators. This incomplete specification may impact the assessment of feasibility success across sites.

#### Implications of the feasibility for the design of the main study

The findings of the feasibility studies offer valuable insights that should be considered to enhance the design and implementation of the forthcoming main studies in the six RECETAS intervention sites. By incorporating these recommendations, the likelihood of successfully conducting the main studies is significantly increased.

The recruitment strategy implemented in the feasibility studies is planned to be replicated in the main study. However, some modifications will be considered to increase the reach of the recruitment process, especially in Marseille. The strategy to advertise and promote the study will be expanded in some intervention sites (e.g., the study will be advertised in local newspapers, radio, and television, and it will be promoted at local events). Nevertheless, the most important take-home message learned from the feasibility studies is that good cooperation and engagement between the research teams and the partners in the community organizations are the key to successful recruitment.

Additionally, some strategies are suggested to retain participants in the main study. First, study personnel need to adhere to the inclusion criteria and consider the inclusion of participants who might hinder their participation in groups. Second, participants should be properly informed about the study including the research dimension of their participation: understanding the possibility of being enrolled in the control or intervention arm (when randomization applies) and the time required for the assessments at the different timepoints. For the participants in the control arm, study personnel should properly manage the participants’ potential frustration when joining a group that does not conduct group activities in nature.

Other strategies that could improve recruitment procedures and reduce the number of dropouts include the engagement of more staff to support recruitment procedures, having follow-up meetings with key stakeholders to reinforce the relationships needed to support recruitment, creating a recruitment advisory board, or considering financial incentives (if not limited by the ethical committees) to attract and retain participants [28].

Regarding the implementation of the interventions, lessons learned from the feasibility studies suggest that it is important not only to create a trusting environment but also to conduct the study in a comfortable and adequate space to facilitate confidentiality when needed. Also, it was found that the assessment usually worked best when led by the researcher, who asked the questions and entered the data, rather than through self-completion. Personalized support might be needed on some occasions because participants might be emotionally affected, and the situation should be properly handled. Since the feasibility is not intended to show improvement, the shorter time period in the feasibility was not a limitation for the main trial, and the key components of the intervention were tested at all sites.

It is important to consider the cultural and social factors of the study population. For example, the ways to express information and opinions among culturally diverse populations may differ also from local terminology to the wording of the instruments used to measure health conditions. Besides that, some participants of the feasibility studies considered certain activities to be demanding or time-consuming. Thus, it is recommended that more information about the activities should be communicated to participants to prevent misunderstandings or unmet expectations related to the level of difficulty of the activities and the length of time required, for example, providing an upfront schedule, informing participants in advance what to bring, and assigning difficulty levels to the activities. Nevertheless, both the feasibility study and the main study should be used to learn what type of activities are more adequate for people, according to different sociocultural and individual factors, to inform healthcare providers, care professionals, social service organizations, and communities for future potential implementation.

## Conclusion

This study provides insights into the design and execution of a feasibility study for complex interventions. It provides valuable information for developing and assessing “Friends in Nature,” a complex nature-based social intervention designed to reduce loneliness and improve health-related quality of life.

Feasibility studies are helpful in assessing practicality, identifying challenges, and informing resource allocation for a project or intervention, ensuring informed decision-making and smoother implementation. The feasibility indicators specifically defined for this study are a useful tool to guide the assessment and interpretation of the feasibility results.

While the feasibility study demonstrated promising outcomes for the implementation of “Friends in Nature” to be considered feasible and acceptable, some challenges were identified that need to be addressed in the main trial. These include minor adjustments to improve (i) recruitment and adherence, (ii) the implementation of the interventions, and (iii) the communication of information related to the activities and health assessments. Addressing these challenges will be crucial for the successful implementation and evaluation of the main studies of the RECETAS project to be conducted in 2023–2024 [14].

## Data Availability

The RECETAS data management plan has established guidelines to inform which data will be open, available upon request, and restricted to project personnel. All open research data in RECETAS will be deposited in a certified repository (e.g., Zenodo), and open access will be established to identify users for access and use of this data. Other resources and tools developed in the project will be made available via the project website (www.recetasproject.eu) or upon request.

## Abbreviation

RCTs: Randomized controlled trials
RECETAS: Reimagining Environments for Connection and Engagement: Testing Actions for Social Prescribing in Natural Spaces
NBSP: Nature-based social prescribing
DJG: 11-item De Jong Gierveld
HRQOL-15: D15-dimensional health-related quality of life
CDR: Clinical Dementia Rating

## Footnotes

1 Duration of the feasibility study per site: Barcelona: 8 weeks, Helsinki: 9 weeks, Prague: 4 weeks, Melbourne: 7 weeks, Cuenca: 9 weeks.
